# Incidence and Molecular Identification of Apple Necrotic Mosaic Virus (ApNMV) in Southwest China

**DOI:** 10.3390/plants9040415

**Published:** 2020-03-28

**Authors:** Wensen Shi, Rundong Yao, Runze Sunwu, Kui Huang, Zhibin Liu, Xufeng Li, Yi Yang, Jianmei Wang

**Affiliations:** Key Laboratory of Bio-Resources and Eco-Environment of Ministry of Education, College of Life Sciences, Sichuan University, Chengdu 610065, China; 2017222045165@stu.scu.edu.cn (W.S.); iamyrd@163.com (R.Y.);

**Keywords:** ApNMV, apple mosaic disease, mosaic symptom, coat protein

## Abstract

Apple mosaic disease has a great influence on apple production. In this study, an investigation into the incidence of apple mosaic disease in southwest China was performed, and the pathogen associated with the disease was detected. The results show that 2869 apple trees with mosaic disease were found in the Sichuan, Yunnan, and Guizhou Provinces, with an average incidence of 9.6%. Although apple mosaic virus (ApMV) is widespread in apples worldwide, the diseased samples were negative when tested for ApMV. However, a novel ilarvirus (apple necrotic mosaic virus, ApNMV) was identified in mosaic apple leaves which tested negative for ApMV. RT-PCR analysis indicated that ApNMV was detected in 322 out of 357 samples with mosaic symptoms. Phylogenetic analysis of coat protein (CP) sequences of ApNMV isolates suggested that, compared with ApMV, ApNMV was closer to prunus necrotic ringspot virus (PNRSV). The CP sequences of the isolates showed the diversity of ApNMV, which may enable the virus to adapt to the changeable environments. In addition, the pathology of mosaic disease was observed by microscope, and the result showed that the arrangement of the tissue and the shape of the cell, including the organelle, were seriously destroyed or drastically changed.

## 1. Introduction

As a member of the *Rosaceae*, the apple (*Malus domestica*) is one of the most widely cultivated fruit trees in the world, with high nutritional value and economic benefits. China is the world’s largest producer of apples, accounting for more than 50 percent of global production in recent years (USDA, 2017). According to the national modern apple industrial system, apple producing areas in China are divided into five regions, namely the Bohai Bay producing area, the Loess Plateau producing area, the Yellow River producing area, the Southwest Cool and High producing area, and the Characteristic producing area, and it is apparent that the Chinese apple production center is moving westward [1].

Up to the 1990s, more than 30 apple virus diseases and related pathogens have been reported in apples worldwide. By 2010, 17 species of apple viruses had been identified in China [2]. Among them, four virus species and one viroid species were common in China, including apple stem pitting virus (ASPV), apple stem grooving virus (ASGV), apple chlorotic leaf spot virus (ACLSV), apple mosaic virus (ApMV), and apple scar skin viroid (ASSVd) [3,4,5,6]. ASPV, ASGV, and ACLSV have not been reported to cause visible symptoms in apple trees, but ASSVd causes severely scarred skin or cracking on the surface of apple fruit, and ApMV produces mosaic leaves on apple trees [3,4,7,8].

Apple mosaic disease was first described by White (1928), Bradford, and Joley (1933), and Christoff (1934) [9,10]. Apple leaves infected with mosaic disease show pale-yellow chlorotic spots or mosaic patterns, which developed along leaf veins or display amorphous chlorotic areas between leaf veins [11]. For a long time, ApMV has been considered the causal agent of apple mosaic disease in major apple producing areas in China. However, several reports have shown that different pathogens may be associated with apple mosaic disease [11,12,13]. From the apple trees which exhibited symptoms of mosaic disease and tested negative for ApMV, a novel ilarvirus (apple necrotic mosaic virus, ApNMV), a close relative of prunus necrotic ringspot virus (PNRSV) and ApMV, was identified by next-generation sequencing analysis in Japan and northern China [11]. The presence of ApNMW was also confirmed in two apple trees showing bright cream spots and mosaic patterns on leaves by RT-PCR, using virus-specific primers in Korea [12]. ApNMV, not ApMV, was the pathogen which causes apple mosaic symptoms in mainland China [13]. Recently, ApNMV was also identified in crabapple (*Malus spp.*) trees in China [14].

From May 2016 to October 2017, an investigation into the incidence of apple mosaic disease in the main apple producing areas in southwest China was performed. In this study, a total of 29,850 apple trees from the Sichuan, Yunnan, and Guizhou Provinces were investigated by visual inspection, and a total of 2869 apple trees with mosaic disease were found, with an average incidence of 9.6%. The leaves of 357 trees displaying apple mosaic were collected from the above three provinces. Total extracted RNAs were subjected to RT-PCR using virus specific primers, and the results revealed the association of ApNMV with the apple mosaic disease in southwest China instead of ApMV, with a positive rate of 90.2%. This is the first report of the ApNMV in southwest China. 

## 2. Results

### 2.1. Apple Mosaic Disease Is Common in Southwest China. 

**Figure 1:**
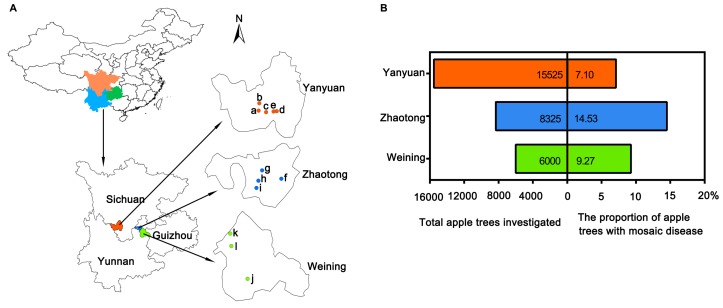
Investigation of apple mosaic disease in southwest China. (**A**) Investigated places in southwest China. Three main apple producing areas in southwest China were shown with different colors, respectively. (**B**) Total apple trees investigated and the proportion of apple trees with mosaic disease.

A total of 29,850 apple trees, including Fuji, Golden Delicious, Red General Fuji, Gala, Zhongqiuwang, Red Star, Qin Guan, and Starkrimson Delicious, were inspected randomly in orchards of 12 towns in the main apple producing areas of the Sichuan, Yunnan, and Guizhou Provinces (Figure 1A). Among 15,525 apple trees in five towns (Meiyu, Weicheng, Shuanghe, Ganhai, and Xiahai) in Yanyuan county, Sichuan Province, 1103 displayed symptoms of mosaic disease, with an infection rate of 7.10% (Figure 1B). Among 6000 apple trees in three towns (Zhongshui, Heishitou, and Niupeng) in Weining county, Guizhou Province, 556 apple trees showed mosaic symptoms, accounting for 9.27%. Among 8325 apple trees in four towns (Sayu, Leju, Sujiayuan, and Shizhahe) in Zhaotong city, Yunnan Province, 1210 apple trees with mosaic disease were confirmed, with the incidence of 14.53% (Figure 1).

### 2.2. Pathogen Detection of Apple Mosaic Disease by RT-PCR 

**Figure 2:**
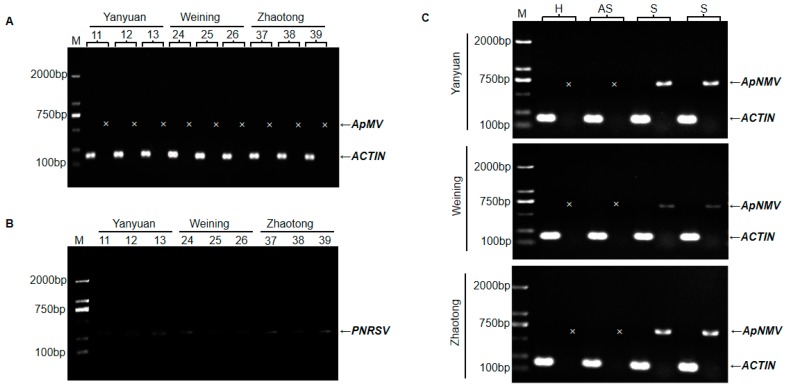
Identification of apple mosaic virus (ApMV) (**A**), prunus necrotic ringspot virus (PNRSV) (**B**), and apple necrotic mosaic virus (ApNMV) (**C**) by RT-PCR. (**A**) Detection of ApMV. Numbers 11/12/13, 24/25/26, and 37/38/39 indicate samples collected from Yanyuan county, Weining county, and Zhaotong city, respectively. (**B**) Detection of PNRSV. Samples are the same as (A). (**C**) Detection of ApNMV. H, healthy leaf as a negative control. AS, asymptomatic leaves from trees with mosaic leaves. S, symptomatic leaves from trees with mosaic leaves. Each sample has two lanes, the left one indicates the product of ACTIN, and the right one corresponds to ApMV or ApNMV. Arrows indicate position of RT-PCR products of ApMV, ApNMV, and ACTIN, severally. M, DNA size marker. The mark ‘x’ indicates none of band at corresponding position. All primer pairs used here were listed in Table S2.

Seven virus-specific primer pairs (Table S1), were utilized to detect the ApMV virus in all leaf samples. ApMV amplicons were never obtained, although the RT-PCR using the primers of ACTIN F/R, which was set as a positive control for amplification of the plant mRNA, resulted in the expected product (Figure 2A). In addition, RT-PCR assays for newly reported cucumber mosaic virus (CMV) and PNRSV in apple [15,16] were performed, and weak bands were detected for PNRSV in most mosaic samples (Figure 2B). Recently, a new virus, ApNMV, that could also cause apple mosaic disease, was reported in Japan and northern China [11]. To determine if the mosaic disease in southwest China was associated with ApNMV, and to further determine the relationships between mosaic leaves and the distribution of the mosaic virus, the specific primer pair (ApNMV-F/R) was synthesized and utilized to amplify ApNMV in the asymptomatic leaves and symptomatic leaves from mosaic trees. Additionally, healthy leaves were included as a negative control. As shown in Figure 2C, ApNMV was detected in symptomatic leaves, but was not detected in asymptomatic leaves from the same branch. Of the 357 mosaic samples tested, 322 were identified as positive, with a positive rate of 90.2%. In 30 asymptomatic leaves samples from mosaic trees, RT-PCR detection yielded negative results.

### 2.3. The Taxonomic Status of ApNMV Isolates in Southwest China. 

**Figure 3:**
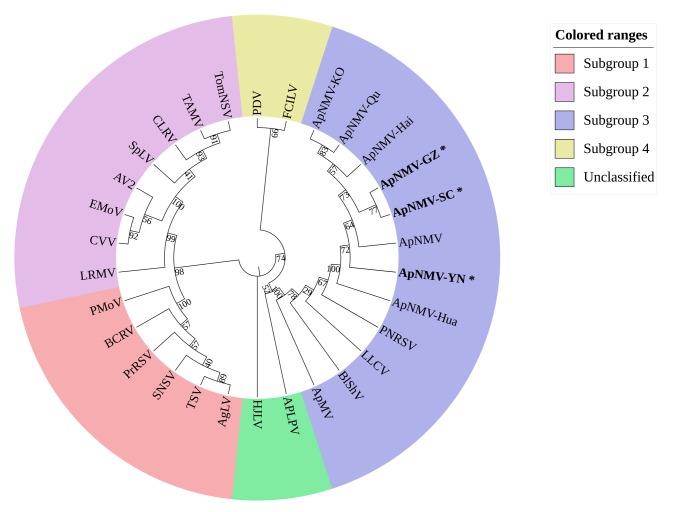
Phylogenetic tree based on the coat protein (CP) amino acid residues of ApNMV isolates with members of the genus ilarvirus. Subgroup 1: Ageratum latent virus (AgLV, NC022129), tobacco streak virus (TSV, NC003845), strawberry necrotic shock virus (SNSV, NC008706), blackberry chlorotic ringspot virus (BCRV, NC011555), privet ringspot virus (PrRSV, NC027930), parietaria mottle virus (PMoV, NC005854). Subgroup 2: asparagus virus 2 (AV2, NC011807), citrus leaf rugose virus (CLRV, NC003546), citrus variegation virus (CVV, NC009536), elm mottle virus (EMoV, NC003570), spinach latent virus (SpLV, NC003810), tomato necrotic streak virus (TomNSV, KT779206), tulare apple mosaic virus (TAMV, NC003835). Subgroup 3: apple mosaic virus (ApMV, NC003480), blueberry shock virus (BlShV, NC022252), lilac leaf chlorosis virus (LLCV, NC025481), prunus necrotic ringspot virus (PNRSV, NC004364). Subgroup 4: fragaria chiloensis latent virus (FClLV, NC006568), prune dwarf virus (PDV, NC008038). No subgroup determined: American plum line pattern virus (APLPV, NC003453), humulus japonicus latent virus (HJLV, NC006066). ApNMV isolate from Japan (P129, LC108995), ApNMV isolate from Korea (KO-276940, LC108995), ApNMV isolate from Shandong Province, China (ApNMV-Hai, ApNMV-Hua, and ApNMV-Qu, MG924900, MG924901, and MG924902), ApNMV isolate from Yunnan Province, China (ApNMV-YN), ApNMV isolate from Guizhou Province, China (ApNMV-GZ), and ApNMV isolate from Sichuan Province, China (ApNMV-SC) were shown.

To determine the taxonomic status of ApNMV isolates in southwest China, a phylogenetic tree based on the amino acid residues of the coat proteins (CPs) of ApNMV isolates and 22 members of the genus ilarvirus, was constructed by neighbor-joining method. As shown in Figure 3, all of the ApNMV isolates, including the ApNMV isolates from southwest China (ApNMV-YN, ApNMV-GZ, ApNMV-SC), the ApNMV isolate from Korea (ApNMV-KO), and the ApNMV isolates from crabapple (ApNMV-Qu, ApNMV-Hai, ApNMV-Hua), together with the ApNMV reported by Japenese, were gathered in the same cluster and were more closely related to PNRSV, LLCV, BlShV, and ApMV in the third subgroup, but far from the evolution of other subgroups. For ApNMV isolates in southwest China, ApNMV-SC and ApNMV-GZ were close to each other in the evolutionary relationship, but relatively far from ApNMV-YN.

### 2.4. Diversity Analysis of ApNMV Isolates.

**Figure 4:**
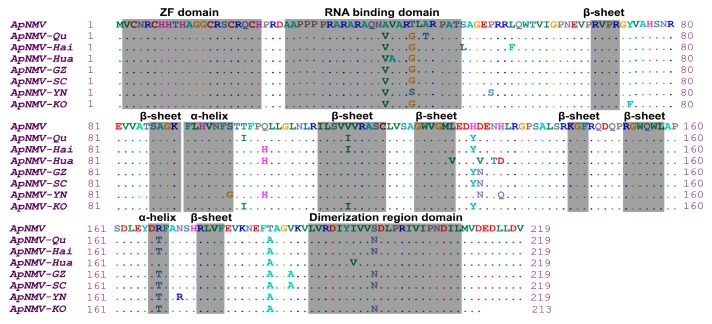
The alignment of eight amino acid residues of CP genes of ApNMV isolates.

ApNMV is a member of genus ilarvirus, comprising tripartite genomes and icosahedral particles [11,13]. RNA3 (its organization shown in Figure S1A) is encoding to movement protein and CP. The CPs of eight ApNMV isolates, with the exception of ApNMV-KO, was composed of 219 aa encoded by 660 nucleotides. In the CPs of ApNMV isolates from southwest China, the amino acid residues of the three isolates shared 95.89% similarity. Compared with the ApNMV identified by the Japanese researchers, the amino acid residues similarity of all isolates ranged from 95.43% to 96.35%. It was predicted by Phyre2 [17] that ApNMV CP has two α-helices and seven β-sheets in the secondary structure, a Zinc finger structure, an RNA-binding domain, and a dimerization region. The predicted structures of the CP gene and the CP of ApNMV are shown in Figure S1B and S1C. Compared with the Japanese isolate, other isolates display a total of 23 aa differences. ApNMV-SC and ApNMV-GZ both had eight aa mutation sites, while ApNMV-YN had ten aa mutation sites. The major difference noted in the predicted protein structure between ApNMV isolates was a difference in the RNA-binding domain at aa position 43, and an α-helix structure at aa position 96 in ApNMV-YN isolates, which was absent in other ApNMV isolates (Figure 4).

### 2.5. Histological and Ultrastructural Observation

**Figure 5:**
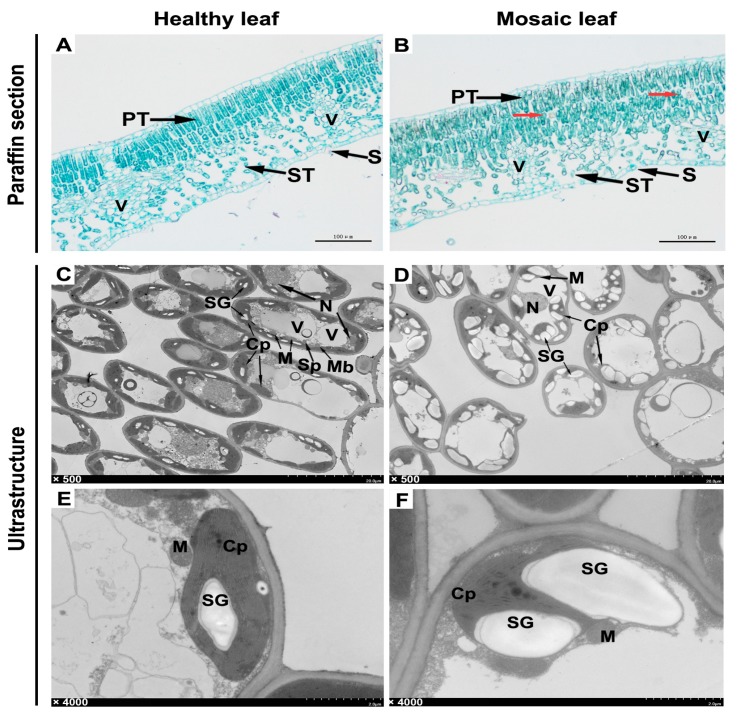
Observation of paraffin section and ultrastructures of leaves. (**A**,**B**) Paraffin section. (**C**–**F**) Ultrastructures. PT, palisade tissue. ST, spongy tissue. V, vascular tissue. S, stoma. Cp, Chloroplast. M, Mitochondrion. N, Nucleus. Sp, Spherosome. Mb, Microbody. SG, Starch Grain. V, Vacuole. The red arrows indicate unidentified granular matter.

Three healthy leaves and three leaves displaying mosaic symptoms were processed into paraffin sections and ultrathin sections for observation under an optical microscope or transmission electron microscope (TEM), respectively. Microstructures showed that in the healthy leaf, the cells of the palisade tissue were long and arranged tightly and neatly, whereas in the mosaic leaf, the cells were relatively short and loosely arranged (Figure 5A,B). Interestingly, some unidentified granular matters were found in the palisade tissue of the mosaic leaf (Figure 5B, red arrows).

Ultrastructures showed that the organelles in cells of the healthy leaf were relatively complete, with spherical vacuoles and fusiform chloroplasts distributed at the edge of cells (Figure 5C). However, the organelles were severely aberrant in the cells of the mosaic leaf, which primarily manifested as abnormally enlarged and shaped vacuoles squeezing the internal structure of the cells, as well as the chloroplasts transforming from fusiform to irregularly spherical (Figure 5D). Further enlarged observation of the internal structure of cells showed that the chloroplasts in the healthy leaves were structurally complete and closely attached to the cytomembrane, and there were fewer starch granules in the chloroplast (Figure 5E). However, the structure of the chloroplasts in the mosaic leaf significantly changed, and the starch granules in them were increased in quantity and shape. At the same time, the chloroplast membrane was severely squeezed or even ruptured (Figure 5F).

## 3. Discussion

Apple mosaic disease is prevalent in apple producing areas worldwide, and has been proved to adversely reduce tree growth and fruit yield [10,18,19,20]. In recent years, mosaic diseases have been reported to occur in the main apple producing areas in China, especially in the Bohai Bay producing area, nevertheless, the information is lacking in southwest China [16,21]. The data provided in this study showed the average incidence of apple mosaic disease in southwest China was 9.6%, well below the previous report of about 25% to 80% in other areas of China [22,23]. Previous research suggested that virus-associated chlorotic or necrotic lesions on leaves are various symptomatic manifestations of host immune responses [24]. In our study, common mosaic symptoms on apple leaves were classified and described, which enriches the types of mosaic disease. It is noteworthy that mosaic leaves were often accompanied by necrotic brown spots. However, it is uncertain whether these spots were caused by ApNMV or other causative agent, including fungi, bacteria, or other viruses. This can be solved if similar symptoms can be observed in pathogenicity studies on the apple seedlings inoculated with infectious clones [13,14,25,26]. Our study reports the most extensive survey to date on the investigation of apple mosaic in southwest China. It will provide new insights to apple mosaic disease and guides to agriculture production. 

After ApMV was not detected in apple mosaic samples, we tried to use the primers of PNRSV, and weak bands were obtained in most mosaic samples. The primer used for the detection of PNRSV was also suitable for ApNMV, based on the sequence and alignment analysis. Meanwhile, neither ApMV nor ApNMV was detected in 35 apple leaves with mosaic symptoms in this study—and it was previously reported that ApNMV was not detected in partial samples having mosaic symptoms [11,13]. It can be concluded from these researches that there may exist other agents except for ApNMV in mosaic apple leaves.

China is the world’s largest producer and exporter of apples, and some apple varieties have also been introduced to other apple-growing countries. Judging from the articles published and our study, ApNMV is widespread in China, the apple trees detected with ApNMV in Japan were introduced from China [11], and only two apple trees were found infected with ApNMV in South Korea, so it is reasonable to suspect that China may be the geographical source of ApNMV. ApNMV has been detected in Asia, including in China, Japan, and Korea [11,12,13]. Symptoms of apple mosaic leaves infected with ApNMV were not distinguishable with symptoms caused by ApMV and PNRSV [10,11]. Whether apple mosaic leaves in other countries are also infected with ApNMV is worthy of further study.

CP is one of the major virulence determinants of viruses [26,27,28]. The result of sequence alignment suggested that the amino acid residues of the CPs of ApNMV exhibited a rich diversity. Compared with Japanese isolate, the others displayed 23 mutation sites in their amino acid residues of CPs, which may enable these ApNMV isolates to adapt to different environments. Although many kinds of apple, including Fuji, Gala, Golden Delicious, and so on, had mosaic leaves, the amino acid residues of the CPs of ApNMV isolates showed no differences between the different kinds of apple from same area in southwest China. The significance of the amino acid residues polymorphism of these ApNMV isolates needs further study.

Previous research demonstrated that mosaic disease could influence plant growth by affecting the photosynthesis of leaves [26,29,30]. In order to uncover how mosaic leaves had been destroyed by pathogens, a mosaic leaf and a healthy leaf from one apple tree were sectioned and observed. Compared with the healthy leaf, the regular arrangement of tissues on the mosaic leaf was destroyed; the cells and their inner organelle were seriously damaged. Notably, the integrity of chloroplast was injured by an abnormal expansion of starch granules. The results were consistent with the reports in tobacco infected with mosaic disease [26,27,31,32]. These findings demonstrate that viruses infecting apple leaves may directly or indirectly impact the normal function of cells and damage to the photosynthesis of apple trees. Apple mosaic symptoms have a strong association with ApNMV. Our previous research indicated that apple leaves collected in southwest China were also infected with ASPV, ASGV, and ACLSV [5]. Whether there are synergistic interactions between these pathogens resulting in the diversity of disease symptoms remains to be further study. It was also found that there were some unidentified granular matters in the palisade tissue of mosaic leaf under microscope view [27]. We are sure that these questions will be of interest to researchers, such as what it is, and how it came to be. 

## 4. Materials and Methods 

### 4.1. Field Investigation and Sample Collection of Apple Orchards in Southwest China. 

From May 2016 to October 2017, a visual survey on the incidence of apple mosaic disease was conducted in the main apple producing areas in southwest China, including in Yanyuan county in the Sichuan Province, Weining county in the Guizhou Province, and Zhaotong city in the Yunnan Province. The total amount of apple trees and the number of apple trees with mosaic diseases were calculated for the incidence rate in each area. A total of 387 apple leaves, including 357 mosaic leaves and 30 asymptomatic leaves from mosaic trees, were collected and stored in a refrigerator at −80 °C for further research. 

### 4.2. The Symptoms of Apple Mosaic Disease. 

**Figure 6:**
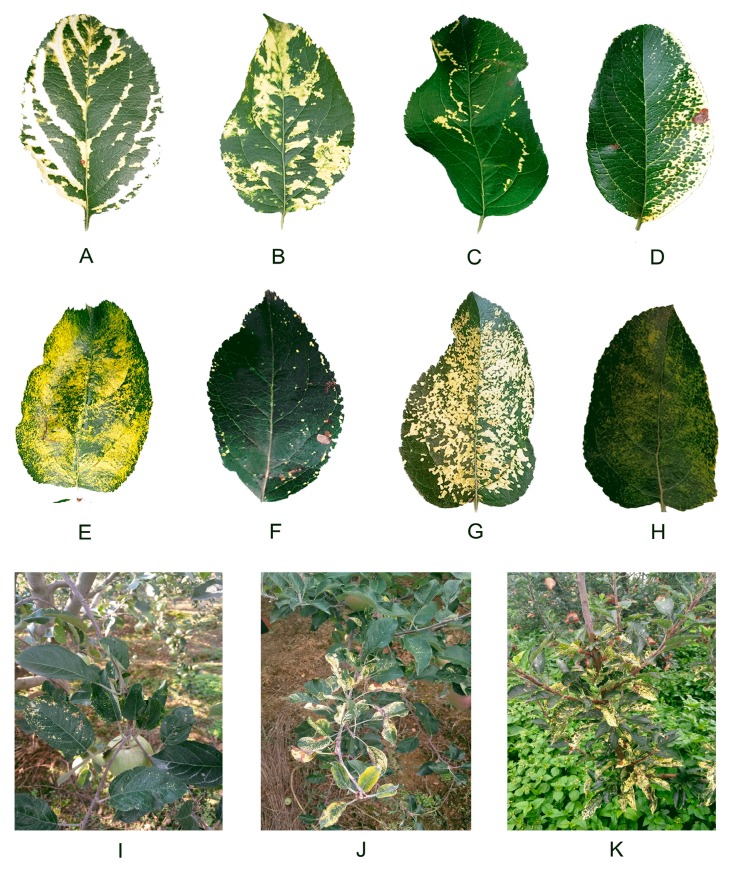
Symptoms of apple mosaic disease. (**A**–**H**) Mosaic leaves. (**A**) striated type, (**B**) plaque-like type, (**C**) annular type, (**D**) secund type, (**E**) painted type, (**F**) limbic type, (**G**) leopard-print type, and (**H**) mottled type. (**I**–**K**) Apple trees with mosaic disease.

Symptoms of apple leaves infected with mosaic disease mainly manifested as flavescent to milky white spots, and bands or plaques with uneven distribution on leaves. According to their morphology, apple mosaic leaves can be divided into the following eight types: striated type (Figure 6A), plaque-like type (Figure 6B), annular type (Figure 6C), secund type (Figure 6D), painted type (Figure 6E), limbic type (Figure 6F), leopard-print type (Figure 6G), and mottled type (Figure 6H), which were described briefly in Table S2. The main types of mosaic leaves in southwest China are the plaque-like type and leopard-print type. Branches and trees with mosaic leaves are also shown (Figure 6I–K). Sometimes, the mosaic disease was accompanied by other diseases, such as brown spot disease (Figure 6F,I). In addition, brownish necrotic spots were apparent (Figure 6D,J,K). 

### 4.3. Total RNA Extraction. 

Each apple leaf sample was fully ground into powder with liquid nitrogen and quickly transferred into a 1.5 ml centrifuge tube containing 500 μL of CTAB extraction buffer (2 M NaCl, 0.1 M Tris-HCI pH 8.0, 20 mM EDTA, 2% CTAB, 2% PVP, 1% β-mercaptoethanol). The mixture was incubated at 65 °C for 10 min, extracted with an equal volume of chloroform/isoamyl alcohol (24:1), and centrifuged for 10 min at 12,000 rpm. The supernatant was removed to a new tube, to which a 1/2 volume of 6 M LiCl solution was added. After a two-hour refrigeration, the mixture was centrifuged at 12,000 rpm at 4 °C for 10 min, the supernatant was discarded, and the pellet was washed with 75% and 100% ethanol once each, and dried for 5 min. Finally, 20 μL of DEPC treated water was added to the pellet to dissolve the nucleic acid, and the solution was stored at −80 °C. 

### 4.4. RT-PCR Detection of ApMV, PNRSV, and ApNMV. 

Total RNAs were extracted from each leaf sample for reverse transcription as described above. The PCR for the identification of the causative agent triggering mosaic disease was performed using the special primer pairs (Table S2). In addition, we used primers of ACTIN F/R for the amplification of the plant mRNA. All PCRs were performed as singleplex reactions. The PCR cycling was 96 °C for 3 min, followed by 34 cycles of 95 °C for 15 s, 57 °C for 15 s, 72 °C for 1 min, and a final extension at 72 °C for 10 min. PCR products were visualized via 1% agarose gel electrophoresis.

### 4.5. ApNMV CP Gene Sequencing

For gene sequencing, total RNAs were reverse transcribed into cDNA, according to the manufacturer’s instructions for the PrimeScript^TM^ RT Reagent Kit (Takara, Japan). Subsequently, the CP genes of the ApNMV isolates were amplified by PCR using a PrimeSTAR Max DNA Polymerase (Takara, Japan) with the specific primer pair ApNMV-F/R. The PCR products were purified and sequenced by the Beijing Genomics Institute (BGI, Beijing, China). Finally, the CP gene sequences of the ApNMV isolates were obtained.

### 4.6. Phylogeny and Diversity Analysis 

MEGA version 7 [33] was used for phylogenetic analysis. The CP amino acid residues of the ApNMV isolates and members of the genus ilarvirus (obtained through the website in reference number [34]) were used to construct phylogenetic trees by neighbor-joining analysis. The bootstrap value was set to 1000 replicates, and the remaining parameters were set by software default. BioEdit 7.0 was used to carry out a homologous sequence alignment on the nucleotide sequence of ApNMV isolate CP and its encoded aa.

### 4.7. Preparation of Paraffin Section and Transmission Electron Microscope Section 

Asymptomatic and symptomatic leaf samples at the same developmental stage were taken from the same growing trees in the same orchard. For histological analyses, samples of mosaic leaves and healthy leaves were soaked in FAA fixative and stored at 4 °C, and then processed into paraffin sections. The sections were dyed by safranine-O for 1–2 h, and were then washed gently with distilled water to remove excess dyes, followed by dehydration with an increasing concentration of alcohol. Subsequently, the sections were stained in a fast green solution for approximately 60 s, dehydrated with anhydrous alcohol three times, and sealed with a neutral gum after clearing with xylene. Finally, the sections were observed under optical microscopes, and images were collected and analyzed. For ultrastructure observations, samples were fixed in 2.5% glutaraldehyde (Servicebio, Wuhan, China). The tissues were rinsed three times with a 0.1 M phosphate buffer (PB, pH 7.4) for 15 min, immersed in a 0.1 M phosphate buffer supplemented with 1% osmic acid (PBS) for fixation at 30 °C for 5 h, rinsed again with PB for 15 min, eluted with gradient acetone, infiltrated by gradient acetone/812 embedding medium (SPI, Westchester, IL, USA), and sliced into 60 nm-pieces after a 48-h drying at 60 °C. The ultrathin sections were double-stained by 1.5% uranyl acetate and lead citrate for 15 min each, dried overnight at room temperature, and observed under a transmission electron microscope (TEM) to collect and analyze images. The experiments were repeated three times. 

## 5. Conclusions

Our results revealed the association of ApNMV with the apple mosaic disease in southwest China instead of ApMV, and the estimation of the incidence of this disease in the main apple producing districts. The molecular characterization and phylogenetic analysis of the CP gene showed the diversity of the local ApNMV isolates. The selection of representative ApNMV isolates in this region that challenge different apple cultivars will help to reveal the most agronomic resistant cultivars in our conditions. The future use of full-length cDNA infectious clones will help to understand plant virus interactions, pathogenicity, and mechanisms of spreading, and will also support host-range studies.

## Supplementary Materials

The following are available online at https://www.mdpi.com/2223-7747/9/4/415/s1, Table S1: Description of different types of apple mosaic leaves. Table S2: primers used for the detection of pathogen. Figure S1. Schematic representation of RNA3 and the predicted structure of CP in ApNMV. (**A**) The position of CP gene on RNA 3 in ApNMV. The dark gray boxes represent the move protein (MP) and coat protein (CP), whereas the light gray boxes represent untranslated regions (Noda H et al. 2017). (**B**, **C**) Predicted secondary structure and functional domain of CP gene (B) and structure of CP protein (C) in ApNMV using online database Phyre2.
