# Supplementary material for: Incidence and Molecular Identification of Apple Necrotic Mosaic Virus (ApNMV) in Southwest China
Source: Plants (Basel). 2020 Mar 28;9(4):415. doi: 10.3390/plants9040415 (PMC7237995; doi:10.3390/plants9040415)
Supplement: Supplementary file 1 [file plants-09-00415-s001.zip › Table S1.docx]

| Primers | Sequences (5'-3') | Size of PCR products | References |
| --- | --- | --- | --- |
| ApMV-F1 | TGGATCTTGCGCTGGATGCA | 570bp | Noda H et al. 2017 |
| ApMV-R1 | ACATTCGTCGGATTTTGCAC |  |  |
| ApMV-F2 | ATGACAACACTGGGAGATAAAC | 860bp | Lakshmi V et al. 2011 |
| ApMV-R2 | TCATCCGCTTATATTTCCAATG |  |  |
| ApMV-F3 | TCGATCGATTCCTTTGTA | 1011bp | Valasevich N et al. 2015 |
| ApMV-R3 | GTTCATCCGCTTATATTTCCATTG |  |  |
| ApMV-F4 | CAAGCGAACCCGAATAAGG | 491bp | Liang PB et al. 2016 |
| ApMV-R4 | ATCACGTACAAATCCCTCAT |  |  |
| ApMV-F5 | CAACCGAGAGGTTGGCA | 206bp | Candresse T et al. 1998 |
| ApMV-R5 | TTCTAGCAGGTCTTCATCGA |  |  |
| ApMV-F6 | CGTGAGGAAGTTTAGGTTG | 417bp | Yardimci N et al. 2008 |
| ApMV-R6 | GCCTCCTAATCGGGGCATCAA |  |  |
| ApMV-F7 | GGCCATTAGCGACGATTAGTC | 668bp | Petrzik K et al. 2002 |
| ApMV-R7 | ATGCTTTAGTTTCCTCTCGG |  |  |
| CMV-F | GATAAGAAGCTTGTTTCGCG | 322bp | Hu Y et al. 2016 |
| CMV-R | GCTCGATGTCGACATGAAGT |  |  |
| PNRSV-F | GAACCTCCTTCCGATTTAG | 357bp | Liang PB et al. 2016 |
| PNRSV-R | GCTTCCCTAACGGGGCATCCAC |  |  |
| ApNMV-F | CTTGCGTGCAATCGATATGG | 736bp | Noda H et al. 2017 |
| ApNMV-R | TCATCTCAACCTAGACATCC |  |  |
| ACTIN-F | GGATTTGCTGGTGATGATGCT | 178bp | This study |
| ACTIN-R | AGTTGCTCACTATGCCGTGC |  |  |

**Table S1**: Primers used for the detection of pathogen.
