# Supplementary material for: Incidence and Molecular Identification of Apple Necrotic Mosaic Virus (ApNMV) in Southwest China
Source: Plants (Basel). 2020 Mar 28;9(4):415. doi: 10.3390/plants9040415 (PMC7237995; doi:10.3390/plants9040415)
Supplement: Supplementary file 1 [file plants-09-00415-s001.zip › Table S2.docx]

**Table S2**: Description of different types of apple mosaic leaves.

| Types | Symptoms |
| --- | --- |
| Striated type | Symptoms occur mainly around secondary veins and chlorotic tissues with milky white. |
| Plaque-like type | Plaques with irregular shape and varisized dimensions are presented on the foliage. |
| Annular type | Atypical annulus or curves are manifested on leaves. |
| Secund type | Speckles mainly emerge on one side of the leaves. |
| Painted type | Serried oily yellow speckles aggregate on the surface of the leaves. |
| Limbic type | Speckles distribute chiefly on the edge of the leaves, forming intermittently yellow borders. |
| Leopard-print type | Dense speckles on the leaves are represented like leopard-print. |
| Mottled type | The leaves are mixed with patches or speckles of faint yellow, forming irregular patterns. |
