# Supplementary figures and images for: Incidence and Molecular Identification of Apple Necrotic Mosaic Virus (ApNMV) in Southwest China
Source: Plants (Basel). 2020 Mar 28;9(4):415. doi: 10.3390/plants9040415 (PMC7237995; doi:10.3390/plants9040415)

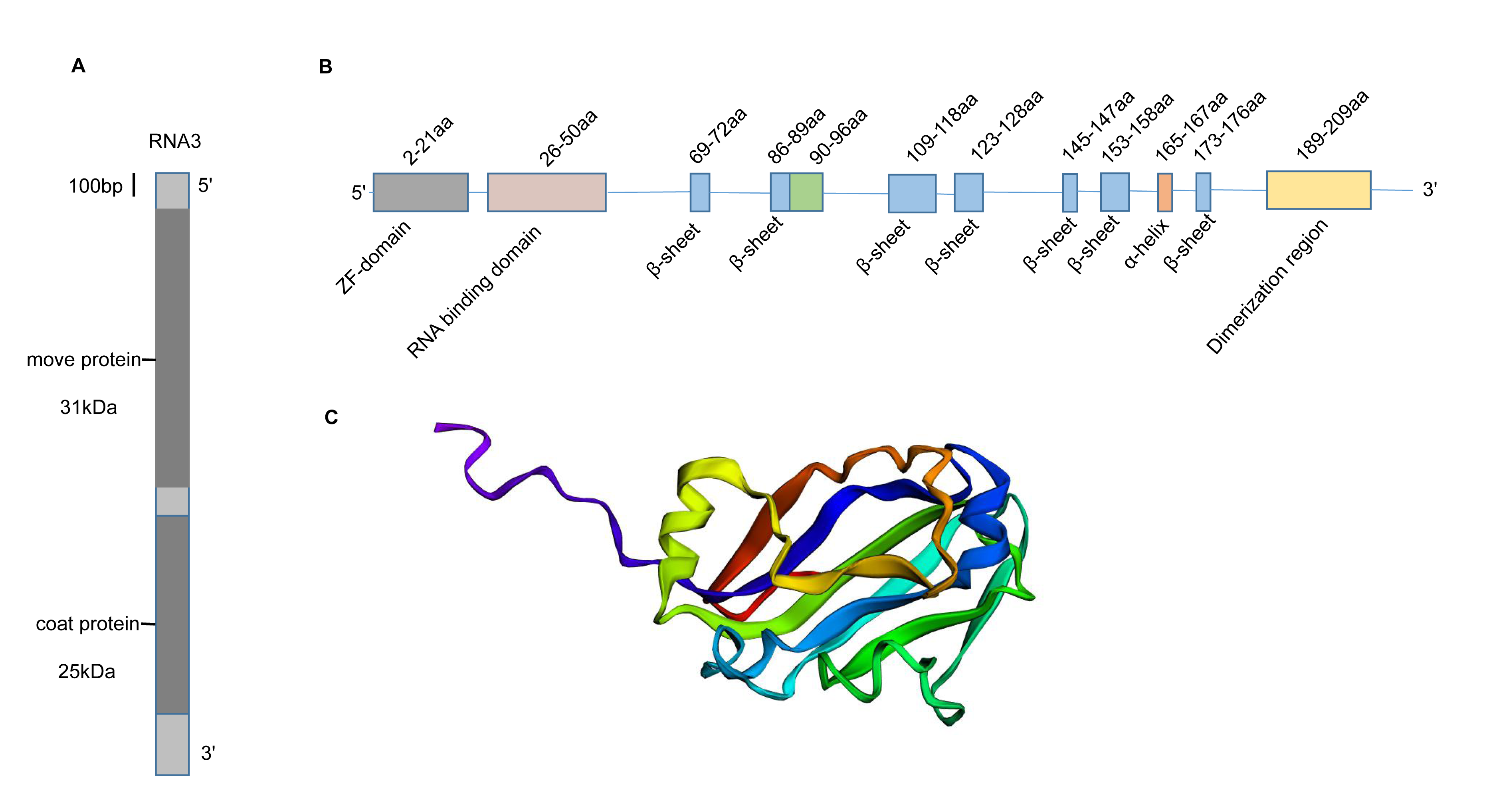

Supplement: Supplementary file 1 [file plants-09-00415-s001.zip › Figure S1.tif]
